# Editorial Comment: Analysis of the Current Surgical Anatomical Knowledge of Radical Prostatectomy: An Updated Review

**DOI:** 10.1590/S1677-5538.IBJU.2026.9909

**Published:** 2026-03-29

**Authors:** Luciano A. Favorito

**Affiliations:** 1 Universidade do Estado do Rio de Janeiro Unidade de Pesquisa Urogenital Rio de Janeiro RJ Brasil Unidade de Pesquisa Urogenital - Universidade do Estado do Rio de Janeiro - Uerj, Rio de Janeiro, RJ, Brasil; 2 Hospital Federal da Lagoa Serviço de Urologia Rio de Janeiro RJ Brasil Serviço de Urologia, Hospital Federal da Lagoa, Rio de Janeiro, RJ, Brasil

**Asher Mandel^1^, Sneha Parekh^2^, Manish Choudhary^2^, Shuichi Morizane^3^, Coskun Kacagan^2^, Neeraja Tillu^3^, et al.**

^1^Department of Urology, Icahn School of Medicine at Mount Sinai, New York, NY, USA;

^2^Department of Urology, Icahn School of Medicine at Mount Sinai, New York, NY, USA;

^3^Division of Urology, Department of Surgery, School of Medicine, Faculty of Medicine, Tottori University, Yonago, Japan

**Eur Urol. 2026 Feb;89(2):128-139.**

DOI: 10.1016/j.eururo.2025.06.002 | ACCESS: 40544124

## COMMENT

Prostatic anatomy is one of the most important points in prostate cancer surgical treatment. Recently the advent of robotic surgery changes the radical prostatectomy, resulting in better outcomes and faster recovery (1-4). The objective in this paper of Mandel and collegues (5) is to survey the existing peer-reviewed literature encompassing prostate anatomy and to consolidate its teachings to the surgeon trainee. The authors summarizes the key findings into a three-dimensional model, bringing the anatomy to life to help learners visualize the complexity of prostate anatomy. The paper conclude that the prostate is a dense organ with muscular and glandular features. Located within the true pelvis, the prostate is surrounded by several fascial planes that contain the neurovascular structures. Lumbosacral parasympathetic nerve root fibers of the pelvic plexus run posterolateral to the prostate gland in a neural hammock configuration. This anatomic understanding informs the NS technique. Finally, the anterior ligamentous structures play a role in continence and possibly erectile function too. We congratulate the authors for the interesting paper.

## Data Availability

Data Not Provided
